# Attitudes and Practices of Surgeons towards Spilled Gallstones during Laparoscopic Cholecystectomy: An Observational Study

**DOI:** 10.1155/2014/381514

**Published:** 2014-10-29

**Authors:** Ramya Yethadka, Shraddha Shetty, Abhishek Vijayakumar

**Affiliations:** Department of General Surgery, Victoria Hospital, Bangalore Medical College and Research Institute, Bangalore 560002, India

## Abstract

The sequelae of spilled gallstones after Laparoscopic cholecystectomy (LC) and the occurring complications may go unnoticed for a long time and can be a diagnostic challenge. The aim of this survey was to study the knowledge, attitude, and practices of surgeons regarding spilled gallstones during LC. An observational, cross-sectional survey, using a questionnaire based on 11 self-answered close-ended questions, was conducted among general surgeons. Of the 138 respondents only 29.7% had observed a complication related to gallstone spillage during LC. There was varied opinion of surgeons regarding management of spilled gallstones, documenting the same in operative notes and consent. It was observed that there is lack of knowledge regarding the complications related to gallstone spillage during LC. There is need to educate surgeons regarding safe practices during LC to avoid gallstone spillage, early diagnosis, and management of complications. There is need to standardize practice to retrieve lost gallstones to reduce complication and legal consequences.

## 1. Introduction

Laparoscopic cholecystectomy is now the method of choice to treat symptomatic gallstones due to lower associated postoperative morbidity. However, it comes with its own spectrum of complications, the two most unique ones being injury to the biliary tract and spillage of gallstones. The former can be minimized by practice and exercising due care during dissection. The latter, however, presents with consequences after a rather protracted period of time, as a whole range of seemingly unrelated symptoms which take the patient to a GP rather than implicate the laparoscopic surgeon.

The aim of this survey was to study the knowledge, attitude, and practices of residents and staff working in the department of surgery in various hospitals of South India with regard to spilled gallstones during laparoscopic cholecystectomy.

## 2. Methods

This was a cross-sectional study carried out in the month of November, 2013, during a surgical conference which saw surgical residents and staff from several teaching institutes of Karnataka, Tamil Nadu, and Kerala. The questionnaire contained 11 self-answered, close-ended questions which addressed the responder's

- experience with and knowledge of complications due to lost gallstones,
- practices regarding patient information and documentation,
- legal liability of the operating surgeon.

## 3. Results

Of the 138 respondents, 22 (15.9%) were consultants while 116 (84.1%) were residents pursuing their postgraduation in general surgery (Table 1).

### 3.1. Knowledge

The experience of this cohort with complications associated with gallstone spillage during laparoscopic cholecystectomy was only 29.7%. With regard to the incidence of spillage, the majority (61.6%) opined that it was less than 10%. When asked about the duration of follow-up, the majority (76.8%) thought 2 years was sufficiently long (Table 1).

**Figure 1:**
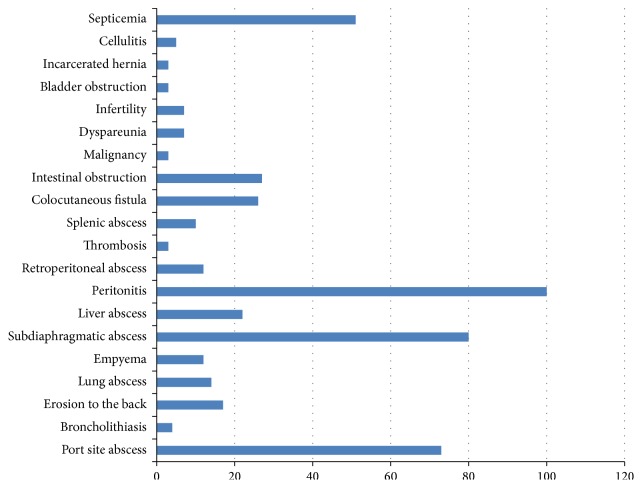
“Which of the following can be complications following gallstones spillage?”

The questionnaire presented a list of 20 possible complications of which the respondents had to pick the ones which could be directly attributed to spilled gallstones. The complications and the responses are presented in Figure 1.

### 3.2. Practices

In case of lost gallstones, 88.4% of the respondents would not convert to an open procedure and would attempt to retrieve the stones laparoscopically (68.1%) or give thorough peritoneal wash and suction (20.3%). 8.7% would convert to an open cholecystectomy whereas 2.9% would rather not do anything.

72.5% of the respondents thought it was necessary to include the risk of spillage and associated complications in preoperative consent but only 48.6% actually did so. 79.7% considered it necessary to mention the lost gallstones in operative notes and 70.3% actually documented the same.

### 3.3. Legal Liability

Only 24% of the respondents had the opinion that the operating surgeon should be held legally responsible for the complications associated with the spilled gallstones.

## 4. Discussion

Laparoscopic cholecystectomy is the gold standard for treating symptomatic gallstones. Perforation of the gallbladder occurs frequently during laparoscopic cholecystectomy and is reported in the range of 10%–40% [1–6]. Gallstone spillage during laparoscopic cholecystectomy is common with the reported incidence of 6%–30% [7–9], which occurs less frequently in open surgery and the spilt stones are easy to retrieve [1]. In our survey all participants acknowledged that stone spillage does occur during laparoscopic cholecystectomy. Eighty-eight percent of participants believed that incidence of spillage was between 0 and 25% which corresponds to the reported incidence. The gallstone spillage can occur during dissection of the gall bladder off the liver bed (75%), tearing with grasping forceps, or during extraction of the gallbladder (25%) through the port sites [10–12]. The predisposing factors for stone spillage are operating on an acutely inflamed gallbladder [1] and the presence of adhesions [4].

Complications that result from these spilled stones are between 0.08% and 0.3% [2, 13].

In our survey majority of participants (70%) did not come across the complications associated with spilled gallstones. Those complications are multiple and widespread; they include abdominal wall abscess [14], broncholithiasis [15–19], lung abscess, empyema [20], erosion to the back [21–23], subdiaphragmatic abscess [24], liver abscess [25], splenic abscess, retroperitoneal abscess [26], peritonitis [27], granulomatous peritonitis, intestinal obstruction [28], thrombosis, colocutaneous fistula [29], malignancy, dyspareunia, and infertility [30, 31], bladder obstruction, incarcerated hernia [32], cellulitis [2], and septicemia [33]. In our survey out of the 21 listed complications only 17% of participants were aware of more than 5 complications. The most common gallstone spillage related complication is abscess formation accounting for 60% of complications [10]. The combination of pneumoperitoneum and peritoneal irrigation disperses calculi within the peritoneal cavity and thus can cause the unusual distant complications.

The time interval between the surgery and the complications of spilled stones varies from as short as one month to as long as 20 years [14, 15] with a peak incidence usually around four months. Seventy-seven percent of participants believed that patients should be on follow-up for the detection of complications for up to 2 years.

The significant risk factors for these complications are acute cholecystitis, spillage of pigmented stones, perihepatic localization of spilled stones, multiple stones (>15) or size (>1.5 cm), and old age [10, 12, 17, 18]. In most instances, the body's immune mechanisms cope, leading to spontaneous resolution. However, infective complications are noticed more often in elderly patients because of poorer immunological reaction. Multiple pigment stones and infected bile increase the incidence of adhesions and intra-abdominal abscesses formation [16].

In our study 72% of participants believed that spillage of gallstone during surgery should be included in informed consent but only 48% were practicing the same. The clear documentation of the intraoperative gallstone spillage in the medical report is recommended for alerting the clinician in the future to the possibility of stones causing any subsequent problems that might lead to earlier diagnosis. In our study around 80% of participants agreed that stone spillage should be mentioned in the operative notes and 70% were practicing the same.

Only a high index of clinical suspicion may lead to correct identification. Ultrasound, computed tomography, and magnetic resonance imaging (MRI) are valuable as diagnostic tools. Ultrasound may identify radiolucent biliary stones in the middle of the inflammatory mass by detecting the hyperechoic acoustic signals from these stones. Ultrasound is more sensitive in detecting stones in abscesses compared with MRI [19] because with MRI it is difficult to differentiate between stones and gas in an abscess. Ultrasound is also more convenient and cost effective.

The prevention of complications is by preventing the stone spillage by careful dissection and use of retrieval bag before extraction of the gallbladder through the port. If gallbladder perforation occurs, the use of suction devices to minimize the spilled bile and spilled gallstones as well as the use of an endobag is mandatory. If possible, the hole in the gallbladder should be closed by the grasp forceps or by an endoclip or endoloop.

Once spillage of stone occurs then every attempt should be made to retrieve all the spilled stones laparoscopically and by performing thorough peritoneal wash with aspiration. Intense irrigation and suction should be performed after collecting the visible stones in order to minimize the number of lost gallstones. This should be done carefully without spreading the gallstones into difficult accessible sites. Stone collection might be facilitated by the use of an intra-abdominal bag and a laparoscopic grasper, a 10 mm suction device, or a “shuttle” stone collector [34]. Conversion at laparoscopic cholecystectomy to retrieve spilled stones is proved to be unnecessary [2]. The use of therapeutic antibiotics in cases of spilled gallstones is only necessary in cases of acute cholecystitis with visibly infected bile or in patients with a high probability for lost gallstones. In our study 68% of participants agreed that laparoscopic retrieval of the spilled stones should be tried, 20% believed peritoneal lavage with suction should be done, and 9% selected conversion to open procedure.

The treatment of the complication consists of eradication of source of infection. Stones which are the foci of infection in these abscesses and sinuses should be completely removed for cure [14, 35]. Abdominal wall abscess from stones caught at the port site can be dealt with by local drainage and evacuation of the stones. In some cases a simple percutaneous excision of the stones is possible [36]. One case of a percutaneous extraction of gallstones using a minimally invasive urological technique has been described [37]. A CT-guided percutaneous insertion of radio-opaque harpoon under local anesthesia, pointing out towards the calcification focus of the upper gallstone followed by elective laparoscopy for the stone retrieval, has been mentioned in the literature [38]. Intra-abdominal abscesses can be dealt with percutaneously by minimally invasive technique [39] and laparotomy where this technique fails. Computed tomography guided drainage of the pus is first done with a pigtail catheter. A few weeks later the tract is dilated with a dilator system and a nephroscope is passed through it and stones are removed [40].

Smaller stones usually less than 1 cm can often be removed through the nephroscope and using a basket. Larger ones need fragmentation by mechanical means or lithotripsy before attempting removal.

In dealing with a deep seated abscess with a tortuous tract electrohydraulic lithotripsy in association with choledochoscopy is a good alternative [41]. A completion contrast study (abscessogram) is recommended to check for the intactness of the cavity and for any retained stones.

Gallstones causing vesical granulomas resulting in haematuria have been dealt with by cystoscopic excision of the granulomas [42].

## 5. Conclusion

There is a dearth of knowledge regarding the consequences and modes of presentation of complication related to lost gallstone. There are varied practices with regard to management, documentation, and patient information. There is need to educate surgeons regarding safe practices during LC to avoid gallstone spillage, early diagnosis, and management of complications. There is need to standardize practice to retrieve lost gallstones to reduce complication and legal consequences.

## Figures and Tables

**Table 1:** Response to questionnaire on gallstone spillage during LC.

Question	Number	Percentage
Designation		
Consultant	22	15.9
Resident	116	84.1
Incidence		
0–10%	85	61.6
11–25%	37	26.8
26–40%	11	8.0
>40%	5	3.6
Complication seen		
Yes	41	29.7
No	97	70.3
Should gallstone spillage be included in informed consent?		
Yes	100	72.5
No	38	27.5
Gallstone spillage included in informed consent		
Yes	67	48.6
No	71	51.4
Intervention for gallstone spillage		
Convert to open for retrieval	12	8.7
Laparoscopic retrieval	94	68.1
Peritoneal wash and suction	28	20.3
None	4	2.9
Necessary to document gallstone spillage in operative notes		
Yes	110	79.7
No	28	20.3
Document gallstone spillage in operative notes		
Yes	97	70.3
No	41	29.7
Duration of follow-up for gallstone spillage		
2 years	106	76.8
5 years	21	15.2
10 years	10	7.2
20 years	1	0.7
Number of complications identified		
<5	114	82.6
>5	24	17.4
Can operating surgeon be held legally liable for complication following gallstone spillage?		
Yes	33	23.9
No	105	76.1

## References

[1] Schäfer M., Suter C., Klaiber C., Wehrli H., Frei E., Krähenbühl L. (1998). Spilled gallstones after laparoscopic cholecystectomy: a relevant problem? A retrospective analysis of 10,174 laparoscopic cholecystectomies. *Surgical Endoscopy*.

[2] Memon M. A., Deeik R. K., Maffi T. R., Fitzgibbons R. J. (1999). The outcome of unretrieved gallstones in the peritoneal cavity during laparoscopic cholecystectomy: a prospective analysis. *Surgical Endoscopy*.

[3] Diez J., Arozamena C., Gutierrez L. (1998). Lost stones during laparoscopic cholecystectomy. *HPB Surgery*.

[4] Rice D. C., Memon M. A., Jamison R. L. (1997). Long term consequences of intraoperative spillage of bile and gall stones during laparoscopic cholecystectomy. *Journal of Gastrointestinal Surgery*.

[5] Sarli L., Pietra N., Costi R., Grattarola M. (1999). Gallbladder perforation during laparoscopic cholecystectomy. *World Journal of Surgery*.

[6] Kimura T., Goto H., Takeuchi Y. (1996). Intraabdominal contamination after gallbladder perforation during laparoscopic cholecystectomy and its complications. *Surgical Endoscopy*.

[7] Catarci M., Zaraca F., Scaccia M., Carboni M. (1993). Lost intraperitoneal stones after laparoscopic cholecystectomy: harmless sequela or reason for reoperation?. *Surgical Laparoscopy and Endoscopy*.

[8] Fitzgibbons R. J., Annibali R., Litke B. S. (1993). Gallbladder and gallstone removal, open versus closed laparoscopy, and pneumoperitoneum. *The American Journal of Surgery*.

[9] Soper N. J., Dunnegan D. L. (1991). Does intraoperative gallbladder perforation influence the early outcome of laparoscopic cholecystectomy?. *Surgical Laparoscopy & Endoscopy*.

[10] Brockmann J. G., Kocher T., Senninger N. J., Schürmann G. M. (2002). Complications due to gallstones lost during laparoscopic cholecystectomy: an analysis of incidence, clinical course, and management. *Surgical Endoscopy and Other Interventional Techniques*.

[11] Woodfield J. C., Rodgers M., Windsor J. A. (2004). Peritoneal gallstones following laparoscopic cholecystectomy: incidence, complications, and management. *Surgical Endoscopy and Other Interventional Techniques*.

[12] Sathesh-Kumar T., Saklani A. P., Vinayagam R., Blackett R. L. (2004). Spilled gall stones during laparoscopic cholecystectomy: a review of the literature. *Postgraduate Medical Journal*.

[13] Horton M., Florence M. G. (1998). Unusual abscess patterns following dropped gallstones during laparoscopic cholecystectomy. *American Journal of Surgery*.

[14] Yao C.-C., Wong H.-H., Yang C.-C., Lin C.-S. (2001). Abdominal wall abscess secondary to spilled gallstones: late complication of laparoscopic cholecystectomy and preventive measures. *Journal of Laparoendoscopic and Advanced Surgical Techniques Part A*.

[15] Noda S., Soybel D. I., Sampson B. A., DeCamp M. M. (1998). Broncholithiasis and thoracoabdominal actinomycosis from dropped gallstones. *Annals of Thoracic Surgery*.

[16] Yadav R. K., Yadav V. S., Garg P., Yadav S. P., Goel V. (2002). Gallstone expectoration following laparoscopic cholecystectomy. *The Indian Journal of Chest Diseases & Allied Sciences*.

[17] Hanna S. J., Barakat O., Watkin S. (2004). Cholelithoptysis: an unusual delayed complication of laparoscopic cholecystectomy. *Journal of Hepato-Biliary-Pancreatic Surgery*.

[18] Chopra P., Killorn P., Mehran R. J. (1999). Cholelithoptysis and pleural empyema. *Annals of Thoracic Surgery*.

[19] Chan S. Y. Y., Osborne A. W., Purkiss S. F. (1998). Cholelithoptysis: an unusual complication following laparoscopic cholecystectomy. *Digestive Surgery*.

[20] Kelty C. J., Thorpe J. A. C. (1998). Empyema due to spilled stones during laparoscopic cholecystectomy. *European Journal of Cardio-thoracic Surgery*.

[21] Memon M. A., Jenkins H. J., Fitzgibbons R. J. (1997). Spontaneous erosion of a lost intra-abdominal gallstone through the back eight months following laparoscopic cholecystectomy. *Journal of the Society of Laparoendoscopic Surgeons*.

[22] Yamamuro M., Okamoto B., Owens B. (2003). Unusual presentations of spilled gallstones. *Surgical Endoscopy*.

[23] Gretschel S., Engelmann C., Estevez-Schwarz L., Schlag P. M. (2001). Wolf in sheep's clothing: spilled gallstones can cause severe complications after endoscopic surgery. *Surgical Endoscopy*.

[24] Sinha A. N., Shivaprasad G., Rao A. S., Sinha A. (1998). Subphrenic abscess following laparoscopic cholecystectomy and spilled gallstones. *Indian Journal of Gastroenterology*.

[25] Steerman P. H., Steerman S. N. (2000). Unretrieved gallstones presenting as a *Streptococcus bovis* liver abscess. *Journal of the Society of Laparoendoscopic Surgeons*.

[26] Parra-Davila E., Munshi I. A., Armstrong J. H., Sleeman D., Levi J. U. (1998). Retroperitoneal abscess as a complication of retained gallstone following laparoscopic cholecystectomy. *Journal of Laparoendoscopic and Advanced Surgical Techniques A*.

[27] Regöly-Mérei J., Ihász M. (1995). The sequelae of retained or lost stones—a complication of laparoscopic cholecystectomy. *Surgical Endoscopy*.

[28] Tekin A. (1998). Mechanical small bowel obstruction secondary to spilled stones. *Journal of Laparoendoscopic and Advanced Surgical Techniques A*.

[29] Patterson E. J., Nagy A. G. (1997). Don't cry over spilled stones? Complications of gallstones spilled during laparoscopic cholecystectomy: case report and literature review. *Canadian Journal of Surgery*.

[30] Chanson C., Nassiopoulos K., Petropoulos P. (1997). Complications of intraperitoneal lost gallstones. *Schweizerische Medizinische Wochenschrift*.

[31] Pfeifer M. E., Hansen K. A., Tho S. P. T., Hines R. S., Plouffe L. J. (1996). Ovarian cholelithiasis after laparoscopic cholecystectomy associated with chronic pelvic pain. *Fertility and Sterility*.

[32] Rosin D., Korianski Y., Yudich A., Ayalon A. (1995). Lost gallstones found in a hernial sac. *Journal of Laparoendoscopic Surgery*.

[33] Van Mierlo P. J. W. B., De Boer S. Y., Van Dissel J. T., Arend S. M. (2002). Recurrent staphylococcal bacteraemia and subhepatic abscess associated with gallstones spilled during laparoscopic cholecystectomy two years earlier. *Netherlands Journal of Medicine*.

[34] Klaiber C., Metzger A., Saager C. (1992). The “shuttle” stone collector—a new device for collecting lost gallstones in laparoscopic cholecystectomy. *Surgical Endoscopy*.

[35] Cacdac R. G., Lakra Y. P. (1993). Abdominal wall sinus tract secondary to gallstones: a complication of laparoscopic cholecystectomy. *Journal of Laparoendoscopic Surgery*.

[36] Bowen J. C., Brenner H. I., Ferrante W. A., Maule W. F. (1992). Gallstone disease: pathophysiology, epidemiology, natural history, and treatment options. *Medical Clinics of North America*.

[37] Whiting J., Welch N. T., Hallissey M. T. (1997). Subphrenic abscess caused by gallstones “lost” at laparoscopic cholecystectomy one year previously: management by minimally invasive techniques. *Surgical Laparoscopy, Endoscopy and Percutaneous Techniques*.

[38] Van Hoecke M., Lissens P., Vuylsteke M., Verdonk R. (2004). Lost gallstones: a relaparoscopic solution to laparoscopic pollution. *Acta Chirurgica Belgica*.

[39] Albrecht R. M., Eghtestad B., Gibel L., Locken J., Champlin A. (2002). Percutaneous removal of spilled gallstones in a subhepatic abscess. *American Surgeon*.

[40] Zamir G., Lyass S., Pertsemlidis D., Katz B. (1999). The fate of the dropped gallstones during laparoscopic cholecystectomy. *Surgical Endoscopy*.

[41] Campbell W. B., McGarity W. C. (1992). An unusual complication of laparoscopic cholecystectomy. *The American Surgeon*.

[42] Famulari C., Pirrone G., Macrì A. (2001). The vesical granuloma: rare and late complication of laparoscopic cholecystectomy. *Surgical Laparoscopy, Endoscopy and Percutaneous Techniques*.

